# Role of adrenomedullin2/ intermedin in pregnancy induced vascular and metabolic adaptation in mice

**DOI:** 10.3389/fphys.2023.1116042

**Published:** 2023-02-17

**Authors:** Chandra Yallampalli, Ancizar Betancourt, Akansha Mishra, Kathleen A. Pennington, Simone Hernandez Ruano, Moises Tacam, Madhu Chauhan

**Affiliations:** Department of Obstetrics and Gynecology, Baylor College of Medicine, Houston, TX, United States

**Keywords:** Adrenomedullin2, intermedin, pregnancy, vascular-adaptation, metabolic-adaptation

## Abstract

**Introduction:** Adrenomedullin2 (AM2) shares its receptor with Calcitonin gene related peptide and adrenomedullin with overlapping but distinct biological functions. Goal of this study was to assess the specific role of Adrenomedullin2 (AM2) in pregnancy induced vascular and metabolic adaptation using AM2 knockout mice (*AM2* ^−/−^).

**Method** **:** The *AM2* ^−/−^ mice were successfully generated using Clustered Regularly Interspaced Short Palindromic Repeats (CRISPR)/Nuclease Cas nine system. Phenotype of pregnant *AM2* ^−/−^ mice was assessed with respect to its fertility, blood pressure regulation, vascular health and metabolic adaptations and compared to the wild type littermates (*AM2* ^+/+^).

**Results** **:** Current data shows that *AM2* ^−/−^ females are fertile with no significant difference in number of pups/litter compared to the *AM2* ^+/+^. However, ablation of AM2 decreases the gestational length and the total number of pups born dead or that die after birth is greater in *AM2* ^−/−^ mice compared to *AM2* ^+/+^ mice (*p* < 0.05). Further *AM2* ^−/−^ mice exhibit elevated blood pressure and elevated vascular sensitivity for the contractile responses to angiotensin two and higher serum sFLT-1 trigylcerides levels compared to *AM2* ^+/+^(*p* < 0.05). In addition, *AM2* ^−/−^ mice develop glucose intolerance with elevated serum levels of Insulin during pregnancy compared to the *AM2* ^+/+^mice.

**Discussion:** Current data suggests a physiological role for AM2 in pregnancy induced vascular and metabolic adaptations in mice.

## Introduction

Adrenomedullin2 (AM2)/Intermedin (IMD) is a hypotensive peptide discovered in 2004 (Roh et al., 2004; Takei, et al., 2004). AM2 belongs to a unique group of calcitonin (CT)/Calcitonin gene related peptide (CGRP) family of peptide hormones and shares sequence homology with its family peptides AM (28%) and CGRP (<20%). In addition, AM2, CGRP and AM share a common 7TM G-protein coupled receptor (GPCR) known as calcitonin-gene related receptor like receptor (CLR). However, the ligand specificity of CLR is dictated by its hetero-dimerization with a group of receptor activity modifying proteins (RAMPs) in such a manner that, RAMP1 and RAMP3 function for CGRP, RAMP2 or RAMP3 can function for AM and all three RAMPs are capable of forming AM2 receptor with CLR (Roh et al., 2004; Takei, et al., 2004). However, majority of the AM2 effects are shown to involve RAMP2 and RAMP3 (Roh et al., 2004; Hay et al., 2018; Garelja and Hay, 2022). These peptides are important for homeostasis in diverse tissues and have similar but distinct physiological effects (Nelson et al., 2000; Roh et al., 2004). Several reports show involvement of these three peptides in reproductive functions (Caron and Smithies, 2001; Chauhan et al., 2006; Chauhan et al., 2009; Chauhan et al., 2011a; Yallampalli et al., 2013; Yallampalli et al., 2014). Genetically modified mouse model of CGRP, AM and their receptor components have been developed with the reports showing embryonically lethal effect of AM, CLR, and RAMP2 ablation but not in CGRP, RAMP1, and RAMP3 null mice (Shindo et al., 2000; Caron and Smithies, 2001; Shindo et al., 2001; Kurihara et al., 2003; Dackor et al., 2006; Ichikawa-Shindo et al., 2008). Thus, although these peptides have some structural similarities and share a common receptor system, they exhibit distinct but overlapping biological functions (Nelson et al., 2000; Roh et al., 2004). Being a novel member of this group of peptides, the physiological role of AM2 in pregnancy is not clearly understood. AM2 is expressed in several tissues including pituitary, hypothalamus, ovary, placenta, and uterus (Roh et al., 2004; Takei, et al., 2004). We have shown that circulatory and placental expression of AM2 is higher during first trimester suggesting a role in implantation and placental development in human and rat pregnancy (Chauhan et al., 2011b; Havemann et al., 2012). This is supported by our report showing that AM2 promotes the invasive capacity of 1^st^ trimester trophoblast cells (Chauhan et al., 2009; Chauhan et al., 2011a) and that the sensitivity of maternal vasculature for AM2 effects is enhanced in rodents as well as in human pregnancy, suggesting a role in pregnancy induced vascular adaptation (Chauhan et al., 2007; Chauhan et al. 2021; Chauhan et al. 2022).

In addition, potential role for AM2 in metabolic homeostasis is reported showing that AM2 treatment restores high-fat diet–induced early insulin resistance in adipose tissue in mice, AM2-tg mice display improvements in high-fat diet–induced early adipose insulin resistance (Lv et al., 2016), AM2 levels negatively correlate with HOMA of insulin resistance in obese human and that, plasma level of AM2 closely associate with different cardiometabolic diseases (Zhang et al., 2018). We reported earlier that infusion of AM2 receptor antagonist AM2_17-47_ during rat pregnancy causes impaired placental function and feto-placental growth restriction (Chauhan, et al., 2006). Serum AM2 level are higher during pregnancy and its expression is downregulated in circulation and placenta in spontaneous abortion and preeclampsia (PE) (Havemann et al., 2012; Chauhan et al., 2016). Interestingly, AM2 levels are lower in second trimester before onset of clinical symptoms of PE in human (Chauhan et al., 2016). More importantly, our recent study shows that AM2 treatment causes relaxation in segments of omental artery isolated from women undergoing preeclamptic pregnancy (Chauhan et al., 2007; Chauhan et al., 2007; Chauhan et al. 2021) suggesting a potential role in the pathophysiology of hypertensive pregnancies such as PE.

Therefore, the goal of this study was to identify the physiological importance of endogenous AM2 peptide during pregnancy using AM2 knockout (AM2^−/−^) mice generated by CRISPER/CAS9 technology. Pregnancy outcome of AM2^−/−^ mice was assessed with respect to regulation of blood pressure, length of gestation, feto-placental health, and metabolic adaptations and compared with their wild type littermates (WT). To identify the role of AM2 in vascular adaptation, maternal sensitivity to ATII and serum levels of sFLT-1 were assessed and its involvement in pregnancy induced metabolic changes were assessed by analyzing glucose tolerance and serum levels of Insulin and triglycerides.

## Materials and methods

### Animal model and procedures

Animal care and use committee (IACUC) of Baylor College of Medicine (BCM) approved this study. All studies were performed under standard 12:12-h light dark cycles in accordance with ARRIVE guidelines. All mice used were C57BL/6J obtained from Jackson Laboratory (Bar Harbor, ME, United States).

### Generation of CRISPR/CAS9 mediated AM2 knockout mice

Genetically Engineered Mouse Core (GEM) and Mouse Embryonic Stem Cell (BCM mES) Core at Baylor college of Medicine (BCM) generated AM2 knockout mice. Briefly, to generate a null allele of AM2, two single guide RNAs (sgRNAs) were selected by BCM mES Core, flanking the genomic region containing the open reading frame of AM2. DNA templates were produced using overlapping oligonucleotides in a high-fidelity PCR reaction. The two SgRNA targeting sequence used are 5′AGA​AGG​GCT​CCC​CAA​CTG​GT and 3′-CAT​ACC​TTG​GCC​CGA​TTC​TC. The SgRNA/Cas9/ss oligo mixture was microinjected into the cytoplasm of pronuclear stage zygotes from C57BL/6NJ female mice by BCM GEM Core. Mice were genotyped by standard PCR using a three-primer system, a single forward primer shared between two different reverse primers. Two primers approximately 100–200 bases outside the two-sgRNA sites were designed to amplify a smaller deletion amplicon compared to the wild-type amplicon. A separate reaction using the second reverse primer, placed within the predicted deleted interval, was designed to amplify a product in the endogenous allele. Therefore, the 3-primer assay for genotyping included a shared forward primer (P1: 5-CCA​AAC​TGG​TTT​TCC​GCT​GG-3″) and reverse primers unique to the wild type (P2: 5’—CTG​AGG​AGT​TCG​GTC​CAA​CC-3″) and CRISPR deletion allele primer (P3: 5’—TCG​GTG​CAG​ATT​CTA​CAG​CC-3″). Genotyped AM2 knockout mice were backcrossed eight times with C57BL/6NJ mice from Jackson laboratory, strain#005304 before using for the experiments.

### Off-target analysis

BCM MES cell core screened heterozygous AM2 null males for the top five potential off-target sites for each sgRNA, identified using the Wellcome Trust Sanger Institute Genome Editing website. Sequencing primers were designed to amplify larger PCR products, which were Sanger sequenced.

### Fertility assay in female AM2 knockout mice

A group of 8 weeks old *AM2* ^−/−^ female mice (KO, *n* = 8) and *AM2* ^+/+^ female mice (wild type littermates, WT; *n* = 8) were assigned to the fertility study. These mice were crossed (monogamous pairs) with wild type proven breeder males (12 weeks old C57BL/6NJ breeders) from Jackson laboratories. The day of observed copulatory plug was identified *as* pregnancy Day (GD) 0.5. The mating pairs were left in cages for 5 months. The gestational age was recorded at 1^st^ delivery. The number of litters/dam and number of total dead pups were recorded over a period of 5 months.

### Assessment of blood pressure, vascular reactivity and feto-placental weights

Another cohort of 8 weeks old mice [*AM* 2^−/−^ and *AM* 2^+/+^ (*n* = 8/group)] was utilized for assessing blood pressure, vascular reactivity, feto-placental weights and serum analysis in non-pregnant and pregnant state. These mice were anesthetized with a mixture of ketamine (Ketalar; Parke-Davis, Morris Plains, New Jersey) and xylazine (Gemini; Rugby, Rockville Center, New York), and telemetric BP transducers (PA-C10 model; Data Sciences, St Paul, Minnesota) were implanted. During the first 3 days, mice were allowed to fully recover from the surgery followed by mating. The day of copulatory plug was considered pregnancy Day 0.5.

*Blood pressure:* Blood pressure (BP) data was recorded for 72 h before mating (non-pregnant) and recording was resumed on day 14.5 of pregnancy for 72 h. The recordings were performed for 30 s at 10-min intervals using the Dataquest ART data acquisition system (DSI, St Paul, Minnesota).

*Feto-placental weights and tissue collection:* Mice were euthanized following BP measurements, feto-placental weights were recorded, mesenteric arteries were collected for vascular studies, and blood for serum extraction and stored at −80°C until analyzed for sFLT-1 levels.

*Vascular reactivity studies:* Two-millimeter segments of second order mesenteric arteries were cleaned off fat and mounted on a wire myograph (DMT 610M) with 25-m tungsten wires. The preparations were bathed in Krebs solution that was maintained at 37°C (pH −7.4). A mixture of 95% O2 and 5% CO2 was bubbled continuously through the solution. The force was recorded continuously by isometric force transducers and analyzed with PowerLab data acquisition system and Lab Chart 7 software (AD Instruments, Castle Hill, Australia). The arteries were normalized according to the manufacturer’s manual and set to the lumen diameter of d1 = 0.9 x d100, where active force development is maximal. After stabilization of the tone, the vessels were contracted twice with 80 mmol/L of potassium chloride (KCl) for 10 min to enhance reproducibility of responses. The second response to KCl was used as a reference contraction in the final calculations. To evaluate endothelial function, response to a single dose of acetylcholine (10^−6^ M) in vessels that were pre-contracted with phenylephrine (10^−6^ or 3 X10^−6^ M) was determined. Only experiments with intact endothelium were used in the final analysis. After 1 h of equilibration, relaxant responses to CGRP_1-37,_ AM_1-52_ and AM2_1-47_ (10^−10^ to 10^−5^ M) were obtained after pre-contraction of the vessels with norepinephrine (10^−7^ to 10^−6^ M) to produce matching contractions in the study groups. In addition, contractile responses to the angiotensin-2 (10^–10^ to- 10^–6^ M) were determined. After each agent was tested, the vessels were washed with Krebs solution and left to recover for 30 min until they returned to their basal passive tension. The Wire Myograph System-DMT- 610M has an automated normalization function, which is assessed from the “Normalization” menu and allows the vessel to be stretched to a normalized internal circumference by a standardized procedure according to the manufacturer’s protocol after equilibration for 30 min at 37°C. An exponential curve is fitted to the internal circumference pressure data. The procedure defines the lumen diameter (d100) that the artery would have had *in vivo* when relaxed and under a transmural pressure of 100 mmHg.

### Intraperitoneal glucose tolerance test

A separate cohort of 8–10-week-old female *AM2* ^−/−^ mice or their wild type littermates (*n* = 12–15) were fasted for 5h prior to breeding with wild type littermate males and after onset of pregnancy on gestational day (GD) 13.5. Fasting blood was collected for serum isolation followed by intraperitoneal glucose tolerance tests (GTT) as previously described (Pennington et al., 2017). Two ReliOn Prime Blood Glucose Monitoring System meters (Walmart, Bentonville, AR) were used to measure glucose levels. Following IPGTT, dams were euthanized.

### Insulin analysis by enzyme linked immunoassay

Insulin levels were assessed in mice fasted for 5 h for IPGTT using Rat/Mouse Insulin ELISA (EMD Millipore, United States) according to the manufacturer’s instructions. Intraassay CV was <10% and inter-assay CV was <12% (*n* = 12–15).

### Triglyceride’s analysis in serum

Serum triglyceride levels were measured in non-pregnant and pregnant mice fasted for 5 h using the Serum Triglyceride Determination Kit (Sigma-Aldrich, United States). Triglycerides were extracted from serum as previously described (Pennington et al., 2017) and then quantified as per Kit instructions (*n* = 6).

### Drugs

Human Alpha-CGRP_1-37_ and human AM_1-52_ were purchased from Phoenix Pharmaceutical Inc., United States; Human AM2_1-47_ was from Alpha Diagnostic, United States, and ATII and U46619 was purchased from Sigma Aldrich, United States.

### Statistical analysis

All data are presented as mean ± SEM. Relaxation responses to the peptides are expressed as percent relaxation of the initial U46619-induced contraction. The second response to KCL was used as a reference to calculate the percent of contraction achieved by ATII. Concentration response curves of drugs are fitted to a log-logistic sigmoid relation, and Emax (maximal relaxation effect) are calculated by using GraphPad Prism. Repeated measures ANOVA (treatment and time as factors) with a Bonferroni *post hoc* was used for comparisons of dose response curves between groups. For other experiments, comparisons between groups were done by Student’s two tailed *t*-test in Prism. Statistical significance was defined as *p < 0.05*.

## Results

### CRISPR/CAS9 mediated knockdown of AM2 in C57BL/6NJ mice

**FIGURE 1:**
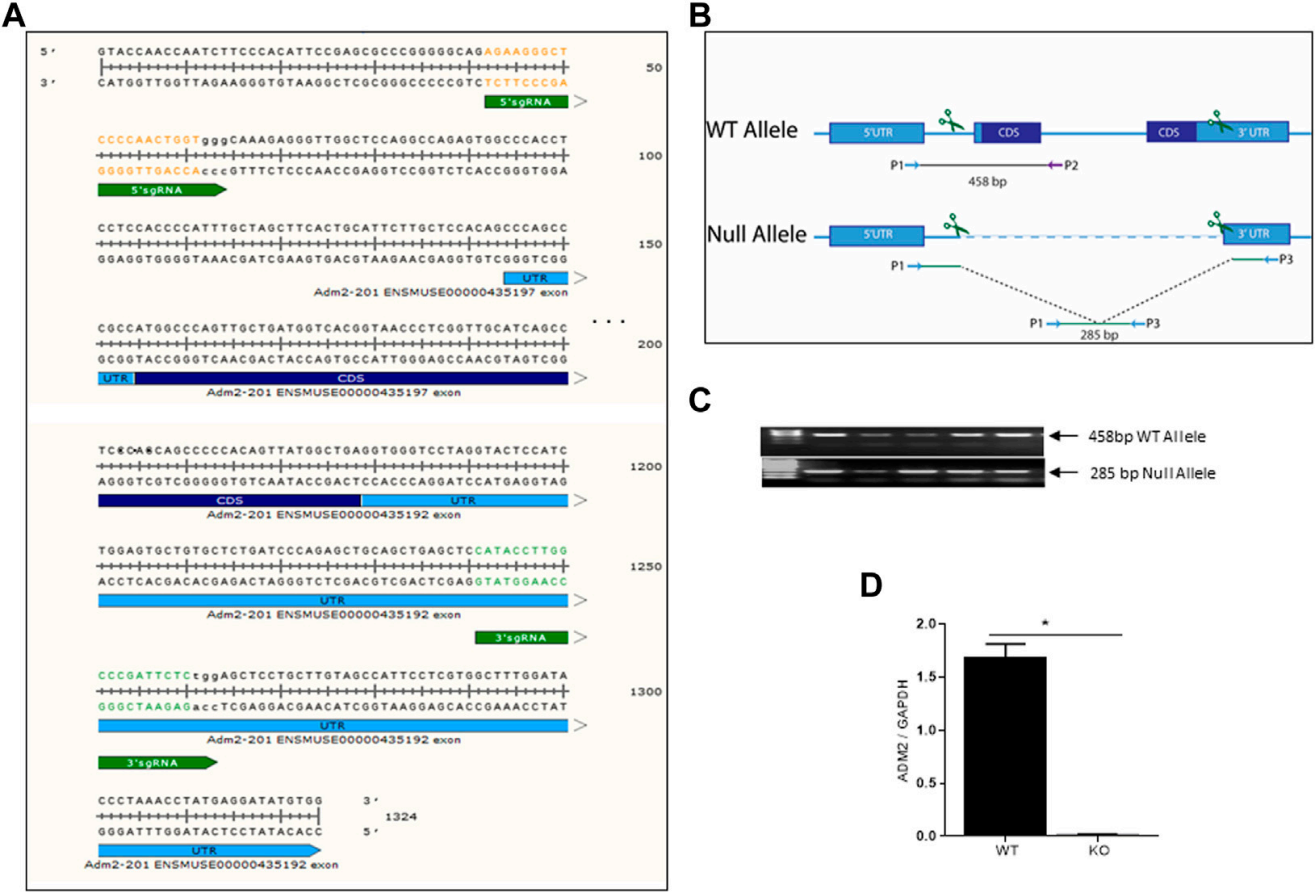
Construction of CRISPR/CAS9 mediated AM2 knockdown: **(A)** Selected sgRNA sites flanking the coding region of AM2 (orange on the 5′ end and green on the 3′end, PAM sites in lowercase), **(B)** Schematic representation of PCR generated wild type allele (458bp) and Null allele (285bp) and **(C)** Representative gel images of 485 bp amplified PCR product for wild type allele (WT) and 285 bp amplified PCR product for null allele (KO), and **(D)** expression of AM2 mRNA in mice placenta. Asterisks (*) indicates significant difference between the groups (*p < 0.05*). Data represents the mean ± SEM, analyzed by unpaired 2-tailed *t*-test.

AM2 knockdown in C57BL/6J mouse was generated by CRISPR/CAS9 technology with the help of Genetically Engineered Mouse core lab at Baylor college of Medicine. Figures 1A shows the two-sgRNA sequences at 5′ and 3’ end of AM2 DNA sequence with the respective PAM sequence spanning the complete full length AM2 gene and Figures 1B demonstrates the PCR assay used for genotyping. The wild type and null allele were genotyped using a 3-primer PCR assay consisting of a shared forward (P1) primer and reverse primer unique to the wild-type (P2) and CRISPR deletion (P3) alleles. Figure 1C shows the PCR product of 458 bp obtained for wild type (WT) allele and 285 bp PCR product obtained for the AM2 knockout allele (*AM2* ^−/−^). Figure 1D shows decreased expression of AM2 mRNA in pla**c**enta of *AM2* ^−/−^ mice compared to their wild type littermates (*p < 0.05*).

### Effect of AM2 ablation on gestational length, pup mortality and pregnancy rate

**FIGURE 2:**
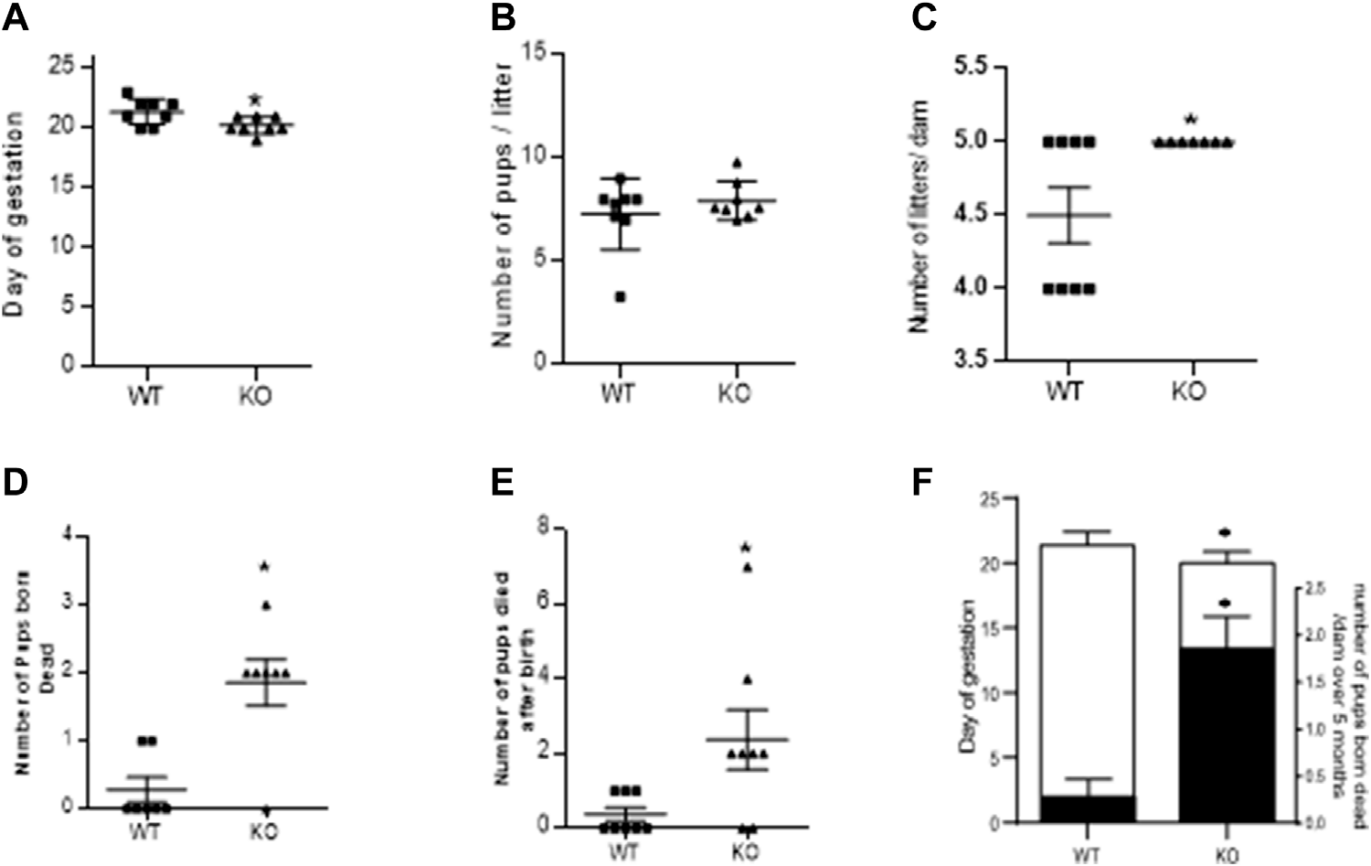
Effect of AM2 ablation on gestational length, pregnancy rate and pup mortality: Figure shows fertility data of *AM2* ^−/−^ (KO) and their wild type littermates (WT) over a period of five consecutive months (n = seven to eight dams/group). **(A)** Day of delivery in 1^st^ pregnancy showing shorter gestational length of KO compared to the WT (*p < 0.05*); **(B)** Mean number of pups born to KO compared to the number of pups born to WT; **(C)** Number of litters born per dam in KO compared to the number of litters per dam in WT over five consecutive pregnancies. **(D)** Mean number of pups born dead in KO compared to the number of dead pups born to the WT (*p < 0.05*); **(E)** Number of pups that died after birth in KO compared to those in WT (*p < 0.05*); and **(F)** Overlapping bar graph showing correlation of decreased gestational age (GA) with increased fetal death (mean number of pups born dead/dam over five consecutive pregnancy Asterisks (*) indicate significant differences between the groups. Data represents the mean ± SEM, analyzed by unpaired 2-tailed *t*-test.

As mentioned in the methods, fertility assay was assessed over a period of five consecutive pregnancies and gestational length was recorded for 1^st^ pregnancy. Figure 2A shows that ablation of *AM2* gene in female mice have shorter gestational length. Figures 2B,C shows that there is no difference in the number of pups/litter or number of pregnancies per dam respectively, over a period of 5 months in *AM* ^−/−^ mice compared to the *AM2* ^*+/+*^. Further, Figures 2D,E show the effect of AM2 ablation on fetal mortality where the mean number of pups that were born dead per dam (Figure 2D) and the mean number of pups that died after birth (Figure 2E) over a period of five consecutive pregnancies is higher in *AM2* ^−/−^ mice compared to those born to the wild type littermates (*p < 0.05*). Therefore, data in Figure 2 suggests that ablation of AM2 results in increased fetal mortality compared to the wild type littermates (Figure 2F) *n* = seven to eight dams/group.

### Effect of AM2 ablation on feto-placental weight

**FIGURE 3:**
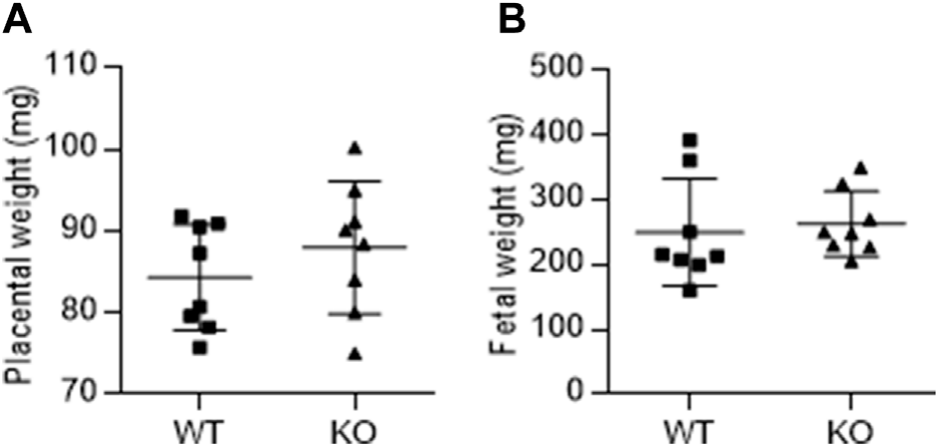
Effect of AM2 ablation on weight of fetus and placenta: Pregnant *AM2* ^−/−^ (KO) mice and their wild type littermates (WT) were euthanized on gestational day 15 and the weights of placenta **(A)** and fetus **(B)** were recorded.

Figure 3 shows the effect of AM2 ablation on the average weight of fetus (Figure 3A) and placenta (Figure 3B) per dam on GD 17.5 of gestation. As shown, AM2 ablation does not affect the weights of fetus or placenta of AM2^−/−^ dams when compared to the wild type littermates (*n* = 8 dam/group; *p > 0.05*).

### Effect of AM2 ablation on blood pressure, serum triglycerides and vascular sensitivity for αCGRP_1-37,_ AM_1-52,_ and AM2_1-47_.

**FIGURE 4:**
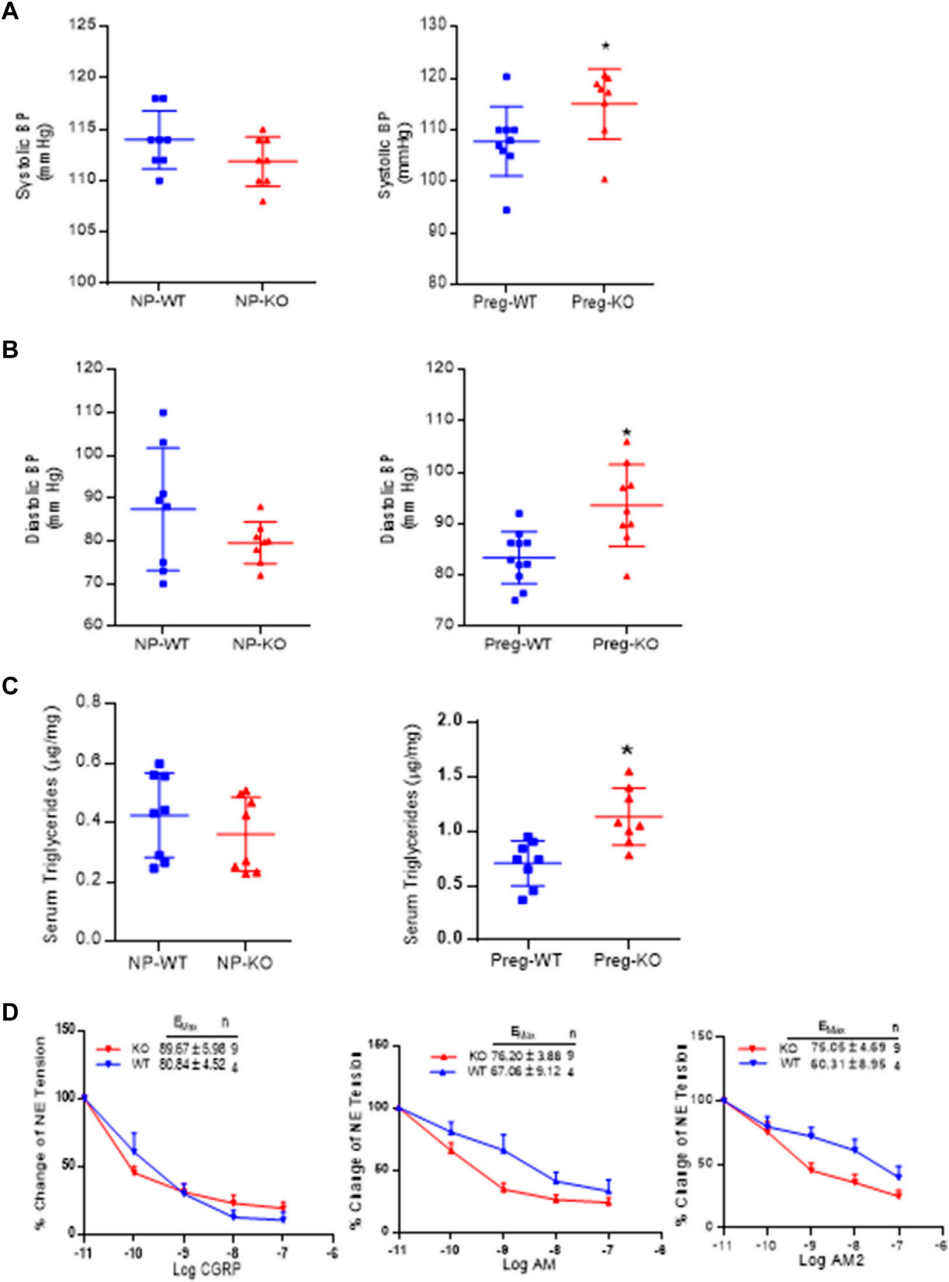
Effect of AM2 ablation on blood pressure, serum triglycerides and vascular sensitivity for CGRP_1-37,_ AM_1-52_ and AM2_1-47_: **(A)** Mean systolic blood pressure recorded for 72 h in non-pregnant [*p > 0.05*] and in pregnant mice [*p < 0.05*] from gestational day 14.5 in *AM2* ^−/−^ (KO) mice during pregnancy compared to their wild type littermates (WT) **(B)** Mean diastolic blood pressure recorded for 72 h in non-pregnant [*p > 0.05*] and in pregnant mice [*p < 0.05*] from gestational day 14 in *AM2* ^−/−^ (KO) mice compared to their wild type littermates (WT); **(C)** Fasting serum levels of triglycerides in non-pregnant (*p > 0.05*) and pregnant AM2 ^−/−^ (KO) mice (*p < 0.05*) fasted on gestational day 13.5 compared to their wild type littermates (WT) and; **(D)** Sensitivity of mesenteric artery pre-contracted with norepinephrine for CGRP_1-37,_ AM_1-52_ and AM2_1-47_ mediated relaxation in *AM2* ^−/−^ (KO) mice compared to the wild type littermates (WT). All data are presented as mean ± SEM, analyzed by unpaired 2-tailed *t*-test and vascular data was analyzed by repeated measures ANOVA (treatment and time as factors) with a Bonferroni *post hoc* for comparisons of dose response curves between te groups. Asterisks (*) indicate significant difference between the groups (*p < 0.05*).

Figure 4A shows the systolic BP in non-pregnant and pregnant mice and Figure 4B is the diastolic BP in non-pregnant and pregnant mice. As shown, ablation of AM2 does not affect the BP of non-pregnant *AM2* ^−/−^ [systolic BP (111 ± 1.9) and diastolic BP (79.60 ± 1.72); *p > 0.05*] compared to the in non-pregnant *AM2* ^+/+^ [systolic (114.0 ± 1.0) and diastolic (79.60 ± 1.20); *p > 0.05*]. Interestingly, during pregnancy blood pressure is elevated in pregnant *AM2* ^−/−^ mice [systolic BP (116.6 ± 1.8) and diastolic BP (93.05 ± 4.42)] compared to the wild type littermates [systolic BP (108.6 ± 3.8; *p < 0.05*) and diastolic BP (83.73 ± 8.73; *p < 0.05*)]. Further, Figure 4C show the effect of AM2 knockout on serum triglycerides in non-pregnant state (*AM2* ^−/−^ [0.3607 ± 0.04428] vs. *AM2* ^+/+^ [0.424 ± 0.0504]; *p* > 0.05, *n* = 8). And in pregnant mice (*AM2* ^−/−^ [1.133 ± 0.093] vs. *AM2* ^+/+^ [0.705 ± 0.073]; *p < 0.05*, *n* = 8). As shown triglycerides in non-pregnant *AM2* ^−/−^ are similar to those in WT mice, However, ablation of AM2 results in increases in serum triglycerides during pregnancy in AM2^−/−^ compared to the *AM2* ^+/+^(*p < 0.05*). Since AM2 is a hypotensive peptide and shares a common receptor system with CGRP and AM, any effect of AM2 ablation on the receptor system was assessed by testing the functional responses of mesenteric artery for relaxation effects of CGRP, AM and AM2. Figure 4D shows the data from wire myograph studies in mesenteric artery segments of pregnant *AM2* ^−/−^ compared to that in pregnant *AM2* ^+/+^ mice. Dose response curves for CGRP_1-37_, AM_1-52_ and AM2 _1–47_ treatments shows that the sensitivity of mesenteric artery for the relaxation effects of CGRP_1-37,_ AM_1-52_ and AM2 _1–47_ in *AM* ^−/−^ mice is similar to that in the *AM2* ^+/+^mice. This is indicative of an intact endogenous CGRP and AM function and presence of a functional receptor system for all three peptides in *AM2* ^−/−^ mice.

### Effect of AM2 ablation on serum levels of soluble fms-like tyrosine kinase (sFLT-1) and vascular sensitivity for angiotensin2

**FIGURE 5:**
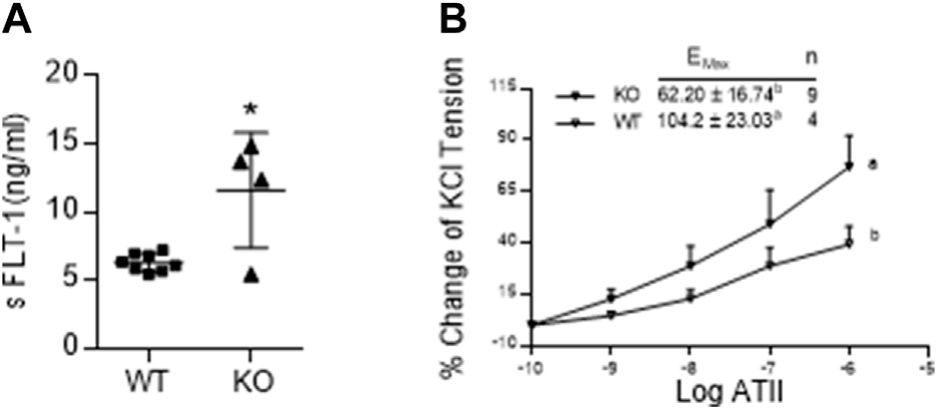
Effect of AM2 ablation on serum levels of soluble fms-like tyrosine kinase (sFLT-1) and vascular sensitivity for angiotensin2: **(A)** Serum levels of sFLT-1 in *AM2* ^−/−^ (KO) and *AM* ^+/+^ (WT) mice on GD 17.5. As shown sFLT-1 levels in *AM2* ^−/−^ mice are significantly elevated compared to the wild type littermates (*p < 0.05*; n = 4–15); **(B)** Dose response curves showing increased contractile effects of angiotensin2 (ATII) [percentage of initial KCL contraction] in *AM2* ^−/−^ (KO) mice compared to the wild type littermates (WT). All data are presented as mean ± SEM, analyzed by unpaired 2-tailed *t*-test (Figure 5A) or repeated measures ANOVA (treatment and time as factors) with a Bonferroni *post hoc* for comparisons of dose response curves between the groups (Figure 5B). Asterisks (*) indicate significant difference between the groups (*p < 0.05*).

Figure 5A shows that the serum levels of sFLT-1 in *AM2* ^−/−^ are significantly elevated in on GD 17.5 compared to the wild type littermates (*p < 0.05*; *n* = 4–15). In addition, dose response curve of wire myograph study in Figure 5B shows that the sensitivity of mesenteric artery for the contractile effects of ATII is significantly elevated in *AM2* ^−/−^ mice compared to the wild type littermates [Emax 104.2 ± 23.03 in *AM2* ^−/−^ vs*.* 62.20 ± 16.74 in AM^+/+^; *p < 0.05*], which is indicative of vascular dysfunction in AM2^−/−^ mice.

### Effect of AM2 ablation on body weight, glucose tolerance and serum levels of Insulin.

**FIGURE 6:**
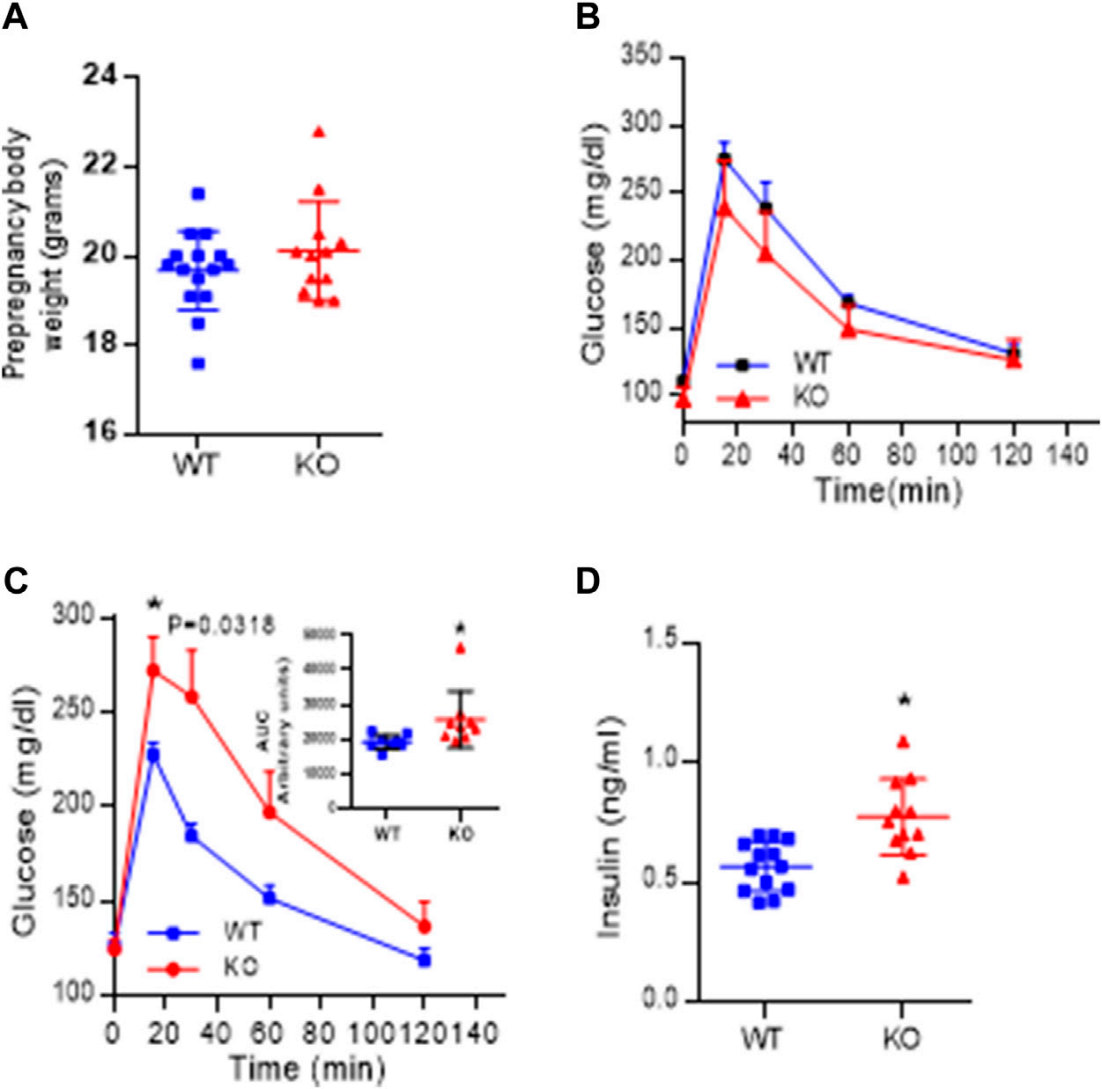
Effect of AM2 ablation on pre-pregnancy body weight, Glucose tolerance and serum levels of Insulin: **(A)** Pre-pregnancy body weight of *AM2* ^−/−^ (KO) mice compared to their wild type littermates (WT) (*p* > 0.05); **(B)** Glucose tolerance test performed in fasted non-pregnant mice before onset of pregnancy in *AM2* ^−/−^ (KO) mice compared to their wild type littermates (WT); **(C)** glucose tolerance test performed in pregnant *AM2* ^−/−^ (KO) mice fasted on gestational day 13.5 compared to their wild type littermates (WT) with area under the curve shown as an inset (*p < 0.05*); and **(D)** Serum levels of insulin in pregnant AM2^−/−^ (KO) mice fasted on gestational day 13.5 compared to their wild type littermates (WT). Asterisk (*) indicate significant difference between the groups with *p < 0.05.* (*n* = 12–15). All data are presented as mean ± SEM, analyzed by unpaired 2-tailed *t*-test.

Body weight recorded before the onset of pregnancy presented in Figure 6A, shows that AM2 ablation does not impact the body weight (*p > 0.05*). In addition, intraperitoneal glucose tolerance test (GTT) performed in non-pregnant mice prior to the onset of their pregnancy presented in Figure 6B shows that ablation of AM2 does not affect glucose metabolism in non-pregnant *AM2* ^−/−^ mice compared to their wild type littermates (*p > 0.05*). However, with onset of pregnancy, *AM2* ^−/−^ mice develop impaired glucose tolerance with increased area under the curve as shown in Figure 6C, *p < 0.05*, compared to the wild type littermates. Further, Figure 6D shows that impaired glucose tolerance is associated with increases in fasting serum insulin levels in AM2^−/−^ mice compared to the wild type littermates (*n* = 12–15, *p < 0.05*).

## Discussion

Current study was designed to assess the physiological role of endogenous AM2 in mice pregnancy using *AM2* ^−/−^ mice with CRISPR/CAS9 mediated systemic ablation of AM2. Data shows that *AM2* ^−/−^ mice are fertile but exhibit increased fetal mortality as assessed over a period of five consecutive pregnancies. In addition, *AM2* ^−/−^ mice exhibit pregnancy induced elevations in blood pressure, serum sFLT-1 levels and AT11 sensitivity along with impaired glucose tolerance associated with elevated levels of insulin and triglycerides in serum. Therefore, this study establishes an important physiological role for AM2 in facilitating vascular and metabolic adaptations in healthy pregnancy and provides a potential mouse model to study pathological pregnancies with vascular and metabolic abnormalities.

Adrenomedullin two facilitates trophoblast invasion in early placental development and circulatory levels of AM2 increase with pregnancy in rodents and humans (Chauhan et al., 2011; Havemann et al., 2012). However, physiological role and importance of AM2 in reproduction is not clearly understood due to its structural similarities with its family peptides, CGRP and AM. In addition, AM2 uses a complex receptor system that is shared by CGRP and AM (Roh et al., 2004; Hay et al., 2018; Garelja and Hay. 2022), which limits the use of AM2 antagonist due to its potential cross-reactivity with other family peptides. Therefore, goal of the current study was to assess pregnancy outcomes of mice in absence of AM2 function. Adrenomedulli2 knockout (*AM2* ^−/−^ mice were successfully generated by CRISPR/CAS9 technology (Figure 1). Data in Figure 2A shows that, in absence of AM2 expression/function, *AM2* ^−/−^ mice are fertile with shorter gestational length. There is no effect of AM2 ablation on the fertility rate (Figure 2B) or litter size (Figure 2C). However, pregnancy in *AM2* ^−/−^ mice suffers with increased pup mortality. The number of pups born dead (Figure 2D), as well as number of pups that die after birth (Figure 2E) is higher in *AM2* ^−/−^ mice compared to the wild type littermates. AM2 is shown to be an estrogen dependent pituitary paracrine factor regulating prolactin release (Lin et al., 2005). As Nursing stimulates prolactin release from the pituitary which promotes continued milk production, it is likely that ablation of AM2 may have affected prolactin release and thus impacted lactation and offspring health resulting in increased pup mortality. Interestingly, unlike our earlier studies showing fetal growth restriction (FGR) in rats infused with AM2 receptor antagonist (AM2_17-47_) during pregnancy (Chauhan et al., 2006)ablation of AM2 in the current study did not affect the feto-placental growth (Figure 3). Instead, although not significant, there was a tendency of higher birth weight in *AM2* ^−/−^ compared to the wild type littermates with very few cases of obstructive delivery necessitating euthanization due to large fetus (observed in only 2 dams (1pup/dam) out of eight different cohorts of pregnant mice and excluded from the study). The discrepancy observed in feto-placental weights in our two studies is likely due to the potential effect of receptor antagonist on the actions of endogenous CGRP and AM function, which are reported to facilitate feto-placental growth during pregnancy (Roh et al., 2004; Yallampalli et al., 2013; Yallampalli et al., 2014). Interestingly, despite the absence of an effect of AM2 ablation on feto-placental weight in *AM2* ^−/−^ mice (Figure 3), pregnancy in these mice resulted in increased fetal mortality compared to the wild type littermates (Figures 2D–F). Although, the cause of increased fetal death in *AM2* ^−/−^ is not clear from this study, it is likely to arise from defective placental development due to impaired trophoblast invasion leading to impaired feto-placental function. This is supported by our earlier reports showing that AM2_1-47_ treatment promotes 1^st^ trimester trophoblast invasion in human pregnancy and its expression coincides with the invasive phase of placental development in rat and human pregnancy (Chauhan et al., 2011b; Havemann et al., 2012).

The primary adaptive mechanism in pregnancy is a marked fall in systemic vascular resistance and a transient reduction in BP along with, pregnancy induced decrease in ATII sensitivity in maternal vasculature (Leal et al., 2022). Adrenomedullin2 is a hypotensive peptide known for its vasodilatory functions (Chauhan et al., 2007; Kandilci et al., 2008; Ross et al., 2010; Hirose et al., 2011). In hypertensive pregnancy with elevated ATII sensitivity, such as PE, AM2 serum levels are downregulated (Havemann et al., 2012; Chauhan et al., 2016). However, it is not known if AM2 has a role in regulating blood pressure and ATII sensitivity in pregnancy. Recent report shows that mice over expressing RAMP1, a co-receptor for AM2 as well as for CGRP, ameliorates ATII mediated hypertension in rodents (Sabharwal et al., 2010). Therefore, due to structural similarities between AM2, CGRP and AM, and overlapping biological function, functional importance of AM2 is not clearly understood in pregnancy. Current study shows that AM2 ablation does not affect the integrity of CLR/RAMPs receptor system as demonstrated by testing the functional response of mesenteric artery for CGRP, AM2 and AM mediated relaxation using wire myograph (as shown in Figure 4D). Sensitivity for the relaxation response of all three peptides is preserved in AM2^−/−^ mice. Ability of CGRP, AM and AM2 to cause relaxation in *AM2* ^−/−^ mice during pregnancy indicate that the CLR/RAMPs receptor system is functional in *AM2* ^−/−^ mice with active endogenous CGRP and AM function. However, although not significant, there was a trend of increased sensitivity for AM2 and AM *in AM2* ^−/−^ mice compared to the AM^+/+^ mice, with AM2 > AM. This may be the compensatory effect of AM ablation in AM^−/−^ mice. In addition, AM2 ablation did not affect the serum levels of triglycerides in *AM2* ^−/−^ compared to the *AM2* ^+/+^ mice in non-pregnant state. Interestingly, despite the intact vascular actions of endogenous CGRP and AM in *AM2* ^−/−^ mice, *AM2* ^−/−^ mice exhibit elevated BP and ATII sensitivity along with elevated serum triglycerides during pregnancy (Figure 4C) compared to their wild type littermates. Therefore, the changes observed in the vascular health of pregnant *AM2* ^−/−^ mice are specific to AM2 ablation and suggest a role for AM2 in maintaining pregnancy induced vascular adaptation. AM2 in paraventricular nucleus (PVN) is reported to attenuate ATII induced generation of reactive oxygen species (ROS) in obese rats with hypertension (Kang et al., 2019). As autonomic nervous system is known to influence peripheral resistance in PE (Schobel et al., 1996), it is likely inhibition/ablation of AM2 function may have an impact on the hypoxia induced increase in the sympathetic activity in PE pregnancy resulting in elevated BP (Quitterer et al., 2004; Baumwell and Karumanchi, 2007).

Further, series of recent studies show that AM2 has protective effect against metabolic syndrome, improving the risk factors for cardiovascular diseases (Zhang H et al., 2016). In human participants, circulating AM2 concentration has been reported to be inversely associated with body weight, BMI, and insulin resistance (Zhang, H. et al., 2016; Zhang S. Y et al., 2016). Further, a recent study showed that AM2 signaling is suppressed in adipose tissue in obesity suggesting lower receptor expression and ligand availability contributing to insulin resistance and other aspects of associated metabolic disorders (Kim et al., 2020). However, ablation of AM2 in the current study did not affect the serum levels of triglycerides (Figure 4C), body weight (Figure 6A) and glucose metabolism (Figure 6B) in non-pregnant *AM2* ^−/−^ mice compared with their wild type littermates. Interestingly, when challenged with pregnancy, *AM2* ^−/−^ mice became developed elevated serum triglycerides (Figure 4C) with impaired glucose intolerance (Figure 6C) accompanied with elevated levels of fasting serum insulin (Figure 6D) compared to the wild type littermates. Stress is a normal reaction to a major physiological change such as vascular and metabolic adaptations that occur in normal pregnancy. This suggest that absence of AM2 function renders increased risk for metabolic disorder under stressful conditions such as establishment of pregnancy. Preeclampsia (PE) and gestational diabetes mellitus (GDM) are common pregnancy complications, occurring only during pregnancy with similar risk factors and patho-physiological changes (Pankiewicz et al., 2022; Yang and Wu, 2022). Evidence from previous studies suggests that the incidence of PE is significantly increased in women with GDM. However, it is not clear whether GDM is independently related to the occurrence of PE or *vice versa*. Current data in pregnant *AM2* ^−/−^ mouse model and published reports on AM2 studies in human pregnancy suggest that PE associated decreases in AM2 levels in human pregnancy may contribute in part to the elevated BP and increased sensitivity of maternal vasculature for vasoconstriction associated with metabolic disorders in pregnancy. However, our earlier reports show that AM2 facilitates trophoblast invasion in 1^st^ trimester and levels of AM2 in amniotic fluid are lower in second trimester of PE pregnancy before onset of the clinical symptoms (Chauhan et al., 2006; Chauhan et al., 2016). Therefore, based on reports and current study, *AM2* ^−/−^ mice appears to represent a mouse model of PE with comorbid gestational diabetes.

## Conclusion

As most of the physiological or pathophysiological effects of AM2 are mediated by CGRP and/or AM receptors, lack of specific antagonists for these receptors sub types impedes the progress in distinguishing AM2 specific action from the overlapping biological functions of its family peptides. This study is the first to show physiological importance of endogenous AM2 in pregnancy induced vascular and metabolic adaptations. This study shows that systemic ablation of AM2 results in increased fetal mortality, elevated BP and serum sFLT-1 levels along with increased vascular sensitivity for ATII. In addition, the vascular defects in *AM2* ^−/−^ mice are associated with impaired glucose metabolism during pregnancy suggesting that inhibition of AM2 function results in vascular and metabolic defects in mice pregnancy. Further, *AM2* ^−/−^ mice can serve as a useful animal model for investigating molecular mechanisms involved in hypertensive pregnancies such as PE, that are associated with metabolic disorders and its consequences on the health of mother and offspring.

## Limitations of the study

Placenta plays a critical role in developmental progression of pregnancy while current study was focused on whole body knockout of AM2. Therefore, future study will assess if the impaired vascular and metabolic function observed in this study are due to the involvement of AM2 in placental functions using mice with placenta specific AM2 knockout.

## Data Availability

The raw data supporting the conclusion of this article will be made available by the authors, without undue reservation.
